# ^125^I Seed Implant Brachytherapy for Painful Bone Metastases After Failure of External Beam Radiation Therapy

**DOI:** 10.1097/MD.0000000000001253

**Published:** 2015-08-07

**Authors:** Shi Feng, Li Wang, Zhang Xiao, Rakesh Maharjan, Li Chuanxing, Zhang Fujun, Huang Jinhua, Wu Peihong

**Affiliations:** From the State Key Laboratory of Oncology in South China, Collaborative Innovation Center of Cancer Medicine, Sun Yat-sen University Cancer Center, Guangzhou, China (SF, LW, LC, ZF, HJ, RM, WP); and Chinese People's Liberation Army General Hospital (301 Hospital), Beijing, China (ZX)

## Abstract

The purpose of this study was to evaluate the safety and therapeutic efficacy of computed tomography (CT)-guided ^125^I seed implant brachytherapy in patients with painful metastatic bone lesions after failure of external beam radiation therapy (EBRT).

From August 2012 to July 2014, 26 patients with painful bone metastases after failure of EBRT were treated with CT-guided ^125^I seed implant brachytherapy. Patient pain and analgesic use were measured using the Brief Pain Inventory before treatment, weekly for 4 weeks, and every 4 weeks thereafter for a total of 24 weeks. Opioid analgesic medications and complications were monitored at the same follow-up intervals.

Before ^125^I seed implantation, the mean score for worst pain in a 24-hour period was 7.3 out of 10. Following treatment, at weeks 1, 4, 8, 12, and 24, worst pain decreased to 5.0 (*P* < 0.0001), 3.0 (*P* < 0.0001), 2.8 (*P* < 0.0001), 2.6 (*P* < 0.0001), and 2.0 (*P* = 0.0001), respectively. Opioid usage significantly decreased at weeks 4, 8, and 12. Overall response rates of osseous metastases after ^125^I seed implantation at 1, 4, 8, 12, and 24 weeks were 58%, 79%, 81%, 82%, and 80%, respectively. Adverse events were seen in 4 patients, including Grade 1 myelosuppression and Grade 1 late skin toxicity.

^125^I seed brachytherapy is a safe and effective treatment for patients with painful bone metastases after failure of EBRT.

## INTRODUCTION

Bone metastasis is a common problem in advanced cancer patient. Based on the latest data, an estimate of 50% of patients with bone metastases will undergo poorly controlled pain during the course of their disease,^1,2^ which affects sleep, diet, emotion, and daily activities, with quality of life severely impaired.^3^ The current management for bone metastasis are primarily palliative, especially for patients with extensive disease involving multiple bone metastatic sites, a systemic approach including chemotherapy, hormonal therapy, bisphosphonates, and radiopharmaceuticals, is used.^4^ External beam radiation therapy (EBRT) is considered the standard treatment for patients with localized uncomplicated painful bone metastases.^5^ However, about 30% of patients do not experience pain relief after EBRT treatment, which may due to the radiation insensitivity of the neoplasm and dose limitation so as to protect the adjacent normal structures.^6^

As an alternative to EBRT, ^125^I seed brachytherapy may resolve this issue by delivering a high dose of radiation directly to the tumor bearing region, while simultaneously sparing adjacent normal tissue. Percutaneous image-guided ^125^I brachytherapy has been a standard method for selected patients with prostate cancer and achieved good local control with few complications.^7,8^ The purpose of our study was to evaluate the safety and efficacy of computed tomography (CT)-guided ^125^I seed implantation in patients with painful metastatic bone lesions after failure of EBRT.

## PATIENTS AND METHODS

### Patients

From August 2012 to July 2014, 26 patients with moderate to severe pain from metastatic bone lesions were recruited into this study, all of whom had prior history of 30 Gy multifraction EBRT or at least of twice 8 Gy single-fraction EBRT to the proposed site of treatment. This study was approved by the ethics committee of the Cancer Center of Sun Yat-Sen University, and each patient provided informed consent. Patients were required to have pathologically confirmed malignant disease and radiographic evidence of bone metastasis. Pain was assessed with the worst pain score from the Brief Pain Inventory (BPI), requiring a score of at least 4 on a scale of 0 to 10.^9^ Patients with up to 2 separate sites of painful metastases were eligible for the study. Previous treatment with bisphosphonates or systemic therapy (chemotherapy, hormonal therapy, and immunotherapy) was not an exclusion criteria for this study. Chemotherapy was not allowed within 3 weeks before or after the ^125^I brachytherapy. The interval between the last fraction of initial radiation and the date of ^125^I brachytherapy had to be at least 4 weeks. The painful site had to be eligible for ^125^I seed implantation with a percutaneous CT-guided approach, this was defined as a location in which seed implantation applicators could be safely placed without significant harm to normal structures. All patients were able to give written consent, and had a life expectancy of greater than 2 months. Patients who had spinal cord compression or a Karnofsky performance status <40 were excluded from the study.

### Pretreatment Preparation

**FIGURE 1:**
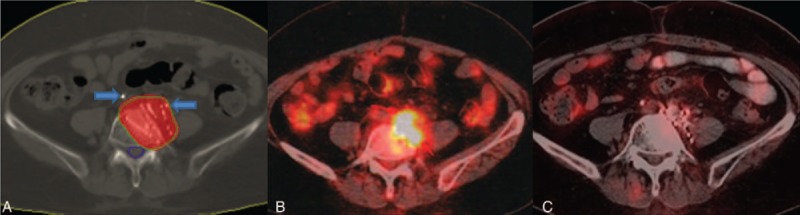
(A) The inner green curve represent the PTV. The blue curve represent spinal cord. The red area is covered by 90% prescribed dose (108 Gy). Arrows point ureteral stents. (B) PET-CT scan before ^125^I implantation treatment. (C) PET-CT scan at 5 months after treatment.

Before ^125^I seed implantation, the effect of focal painful metastatic disease was assessed by use of the BPI. Opioid analgesic medication use was translated into a morphine equivalent dose and recorded.^10^ Before treatment, physical examination of the patients was carried out by a participating radiologist in order to determine the site or sites of focal pain correlated with available imaging including CT, magnetic resonance (MR) and positron emission tomography-computed tomography (PET-CT), which were obtained within 1 month (Figure 1B). Within 1 week of the implantation procedure, a complete blood count and prothrombin time were obtained. The brachytherapy treatment planning system (BTPS, Beijing Atom and High Technique Industries, Inc., Beijing, China) was used to create implant plans based on the CT images. Based on the most recent CT scan, the gross tumor volume (GTV) was clearly identified. Oncologists outlined the prescribed dose target volume (PTV) to cover the lesion with a 0.5 to 1 cm margin. The prescribed dose of radiation was 100 to 120 Gy, which was adjusted according to the previous radiation dose and adjacent structures.

### Treatment Procedure

The ^125^I seed implantation was performed under general anesthesia or moderate sedation at the discretion of the consultant doctor, and under routine CT guidance according to the plan. Local anesthesia by 1% lidocaine was given both intradermally and around the periosteum. For lesions that had destroyed the bone cortex, the 18-gauge implantation needles (Doctor Japan, Gyoda, Saitama, Japan) were placed directly into the metastasis. For lesions with intact cortical bone, 16-gauge coaxial bone biopsy needles (Ackerman, Cook, Bloomington, IN) were placed into the lesions. After the core and the inner trephine needle were removed, the implantation needle was placed through the outer cannula into the metastasis. A median of 48 ^125^I seeds (model 6711, 4.5 mm long and 0.8 mm in diameter, China Institute of Atomic Energy) were implanted.

### Posttreatment Patient Assessment

For each patient, the posttreatment assessment was obtained (Figure 1A). The actuarial D90 (dose delivered to 90% of the target volume) was larger than prescribed dose in all patients and ranged from 104 to 137 Gy (mean 116 Gy). The V100 (the percentage of the target volume receiving at least 100% of the prescription dose) of each patient was more than 95%. Patients were evaluated for severity of pain by using the BPI. The patients completed the BPI with the assistance of a study coordinator with respect to the focal painful metastasis. If 2 metastases were treated, a single response was recorded for the more painful of the 2 treated areas. The BPI was completed at 1, 2, 3, 4, 8, 12, 16, 20, and 24 weeks. Analgesic use was also recorded during each of these interviews. Each patient underwent a contrast-enhanced CT examination or PET-CT of the treated region 3 to 5 weeks after the treatment. Longer follow-up imaging is conducted according to the recommendation of clinician (Figure 1C). A complete response was defined as a BPI worst pain score of zero at the treated site with stable or reducing daily oral morphine equivalent dose. A partial response was defined as a worst pain score reduction of 2 or more at the treated site without an increase in daily oral morphine equivalent dose, or as a reduction of analgesic use of 25% or more from baseline without an increase in pain. Pain progression was defined as an increase in worst pain score of 2 or more above the baseline at the treated site with stable analgesic intake, or as an increase in daily oral morphine equivalent dose of at least 25% with worst pain score stable or one point above the baseline. Indeterminate response was defined as any response not captured by the complete response, partial response, or pain progression definitions.^11^ Overall response rate = complete rate + partial response rate.

### Statistical Methods

This was a single-arm observational study, with patients as their own controls through relevant monitoring. The primary end point was worst pain in a 24-hour period. A 2-score drop in worst pain was defined as clinically significant. We used a 2-sided paired *t* test to infer the sample size, with 5% type I error rate. A sample size of 26 patients would provide 90% power to detect a difference of 2-score on average. Analysis of the worst pain in a 24-hour period was undertaken via paired *t* tests across each time points, supplemented by repeated measures the correlated variance. Findings with *P* values <0.05 were considered statistically significant. The other questions regarding quality of life contained in the BPI were performed using the same method. A Wilcoxon rank-sum test was utilized as confirmatory analyses instead of a *t* test. For missing values, complete case analysis was used and imputation via the mean value, nearest neighbor, last value, and worst value carried forward approaches.^12,13^

## RESULTS

### Patient and Treatment Characteristics

The patient demographics and tumor characteristics are shown in Table 1. There were 26 patients (median age, 49 years) who underwent ^125^I seed implantation for painful bone metastases, including 22 men and 16 women. Out of the 26 treated patients, 12 patients (46%) had previously received bisphosphonates and 24 patients (92%) had previously received opioid analgesics for the painful lesions that were to be treated. Seventeen of 26 (65%) patients had received 30 Gy multifraction EBRT and 9 patients (35%) had received at least of twice 8 Gy single-fraction EBRT to the proposed site of treatment. The size of the treated lesions ranged from 1.8 cm (ischium) to 6.3 cm (sacrum). Lung (n = 5) and liver (n = 5) cancer were the most common types of cancers treated. The anatomical regions treated were the vertebrae, pelvis, rib, scapula, and extremity. Of all the tumors treated, there were 7 tumors with an intact cortex requiring bone needle access before coaxially inserting the 18-gauge implantation needles. Single tumors were treated in 24 patients and 2 tumors were treated in 2 patients. Therefore, a total of 28 tumors were treated in this study.

**TABLE 1:**
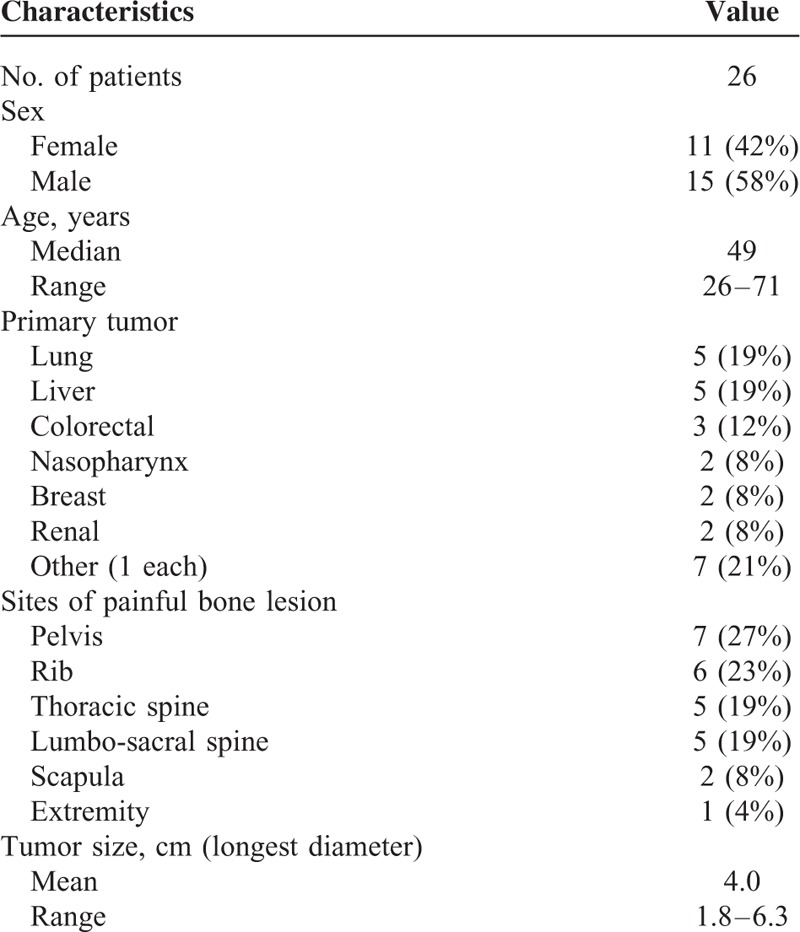
Patient and Tumor Characteristics

### Responses and Complications

**FIGURE 2:**
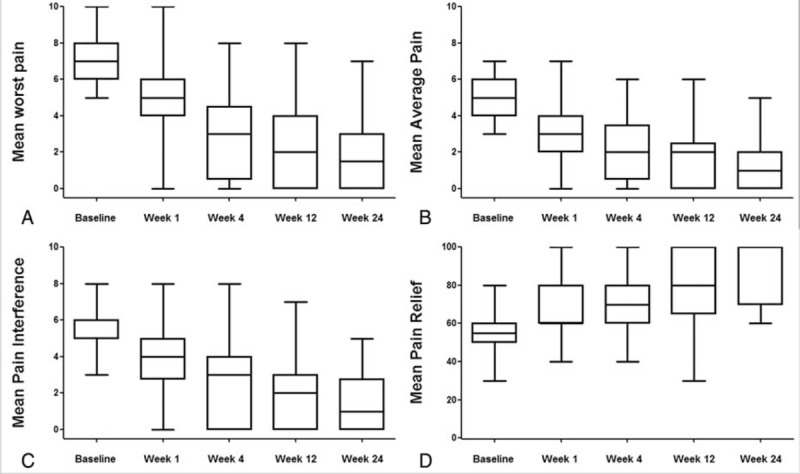
Summary box plots of BPI pain scores over time for patients treated with ^125^I seed brachytherapy. (A) Worst pain. (B) Average pain. (C) Interference of pain in daily activities. (D) Pain relief from ^125^I seed brachytherapy or medications.

The change of worst pain, average pain, pain interference, and pain relief after the ^125^I seed implantation were listed in Table 2. The mean worst pain score in a 24-hour period was 7.3 before ^125^I seed implantation. After treatment, at 1, 4, 8, 12, and 24 weeks, mean worst pain scores decreased to 5.0 (*P* < 0.0001), 3.0 (*P* < 0.0001), 2.8 (*P* < 0.0001), 2.6 (*P* < 0.0001), and 2.0 (*P* = 0.0001), respectively (Figure 2A). Similar decreases in average pain and pain interference were also observed (Figure 2B and C). Pain relief after ^125^I seed implantation improved from 54% at baseline to 66% at week 1 (*P* < 0.0001), 74% at week 4 (*P* < 0.0001), 76% at week 8 (*P* < 0.0001), 80% at week 12 (*P* < 0.0001), and 88% at week 24 (*P* = 0.002) (Figure 2D).

**TABLE 2:**
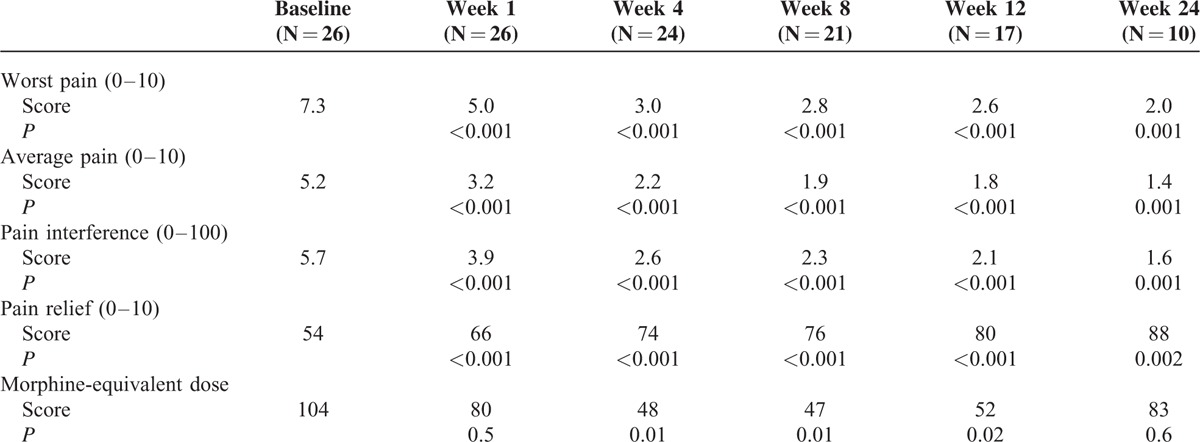
Brief Pain Inventory—Short Form Mean Pain Scores and Opioid Requirements at Baseline and Following ^125^I Seed Brachytherapy

Following ^125^I seed implantation, a reduction of opioid analgesic medications consumption was reported in 83% (20/24 patients) of the patients who use the opioid analgesics before the procedure, and 46% (11/24 patients) reported no opioid analgesic medications at some time during the follow-up. Mean opioid requirements decreased from week 1 (Table 2), and statistically significant reductions in opioid usage were seen by weeks 4, 8, and 12. The opioid usage increased at week 24 in comparison to weeks 4, 8, and 12, although the corresponding site-specific pain scores decrease at that time.

Overall response rates of osseous metastases after ^125^I seed implantation at 1, 4, 8, 12, and 24 weeks were 58%, 79%, 81%, 82%, and 80%, respectively. Patient clinical responses are summarized in Table 3. At week 12, a complete response was observed in 29% (5 patients) of the 17 patients, and a partial response was observed in 53% (9 patients). Only 6% (1 patient) of the 17 patients showed progression of pain.

**TABLE 3:**
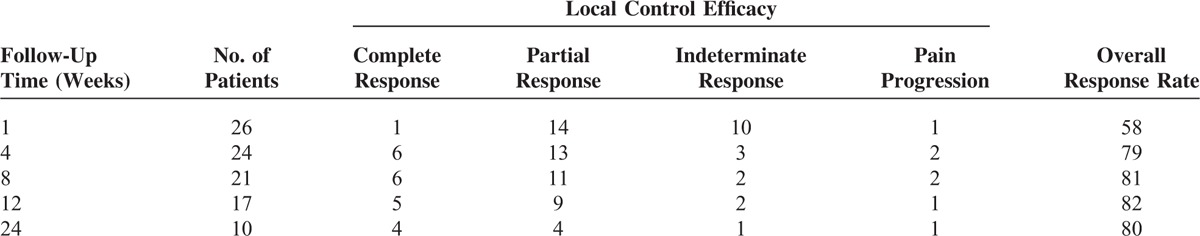
Clinical Efficacy of ^125^I Brachytherapy on Bone Metastatic Tumors

Several complications were related to ^125^I seed implantation. Temporary redness of skin was observed in 3 patients, and a further 1 patients developed temporary skin pigmentation within 8 weeks of seed implantation. One patient was found to have Grade 1 myelosuppression which returned to normal within 12 weeks. No instances of moderate or severe complication due to injury of the spinal cord, major motor nerves, artery, or bowel were recorded.

## DISCUSSION

Bone metastasis is the most common source of moderate and severe cancer pain, and approximately 75% of patients with advanced cancer experience bone pain.^14^ Analgesic effects of EBRT on painful bone metastasis have been known since the beginning of the 20th century. Indications for EBRT for bone metastases include pain, risk for pathological fracture, and neurological complication arising from spinal cord compression or nerve root pain.^15^ However, few options exist for patients after failure in obtaining adequate pain relief following EBRT. The disease of these patients is often insensitive to chemotherapy or hormonal therapy, while surgery is mostly reserved for impending fracture, but not for patients presenting with advanced disease and poor functional status. Opioid analgesics remain the only option for these patients, while their adverse effects such as nausea, constipation, and sedation can be significant.^4^

In our study, highly significant reductions in pain scores and improvement in quality of life following ^125^I seed implantation of painful bone metastases were reported. In total, 85% (22 patients) experienced a complete response or a partial response. The pain relief was rapid, with a 2.3 reduction of mean worst pain week 1, and a 4.3 by week 4. These data are also consistent with change of opioid requirement as similar significant decreases in opioid requirements were observed from week 4. However, opioid requirement increased at week 24, despite continued reductions in pain scores. The increased opioid requirements could be caused by the development of pain at other sites of metastases, despite persistent pain relief at the treated site, since these patients were at the end of their lives.

The irradiated area of EBRT is larger than ^125^I seed brachytherapy, which could potentially lead to severe radiation damage to surrounding normal tissue of bone lesions. Therefore, the dose of EBRT is often limited by the tolerance dose of the normal tissue, leading to incomplete killing of tumor cells, especially those radiation insensitive cells. Moreover, sublethal damage of tumor cells could be repaired after EBRT and proliferate thereafter. According to the BTPS, the therapeutic volume of the ^125^I seed implanted inside the tumor fits the shape of the tumor. Large doses of radiation would be emitted “from inside out” of the tumor and then undergo rapid fall off to the normal tissue since the radioactive energy is inversely correlated with the square of the radius. As a low-dose rate brachytherapy, ^125^I seed has several advantages over EBRT. Firstly, it emits continuous low-dose γ rays that can inhibit tumor cell proliferation^16^; secondly, sustained low-dose radiation reduces repair rates of sublethal damage to tumor cells^17^; thirdly, the slow emission rate of the radiation also gives surrounding normal tissue sufficient time for repair of sublethal damage, protecting healthy organs from late tissue damage.^18,19^ Given these advantages, ^125^I brachytherapy has been successfully used in clinic for the treatment of solid tumors such as prostate carcinoma, glioma, pancreatic cancer, hepatocellular carcinoma, melanoma, and head and neck neoplasms.^18–24^ A BTPS can help peripheral tumor doses reach the prescription dose of 100 to 120 Gy. Our current treatment plan can make more than 95% of the tumor get 100% prescription dose, so that the tumor target receives adequate dose without increasing radiation to surrounding normal tissue.

^125^I seed implantation of metastatic lesions involving bone is a safe procedure. In this study, 4 patients had complications. Grade 1 skin toxicity were seen near the areas of ^125^I seed implantation in 3 patients. One patient with iliac metastases was found to have Grade 1 myelosuppression. No severe complications were observed. This observation coincides with the results of previous studies. Yang et al performed the implantation of the ^125^I seed in the T13 vertebra body of 20 banna pigs after an observation of 8 months. They report no case of radiation myelopathy and no significant cellular impairment.^25^ Lynda et al reviewed the data on 58 patients whose site of ^125^I implant was at close proximity to osseous tissue. There appears to be no excessive osseous toxicity either clinically or radiologically with ^125^I seed implantation, with a follow-up up to 8 years.^26^ Despite the relatively low risk of major complications as reported, careful case selection and the planning of reasonable security paths under CT imaging guidance are also necessary to avoid injury of critical structures such as spinal cord, major motor nerves, artery, and bowel.

Limitations of this study include the heterogeneity of cohort of patients with respect to site, size of the metastasis, and primary tumor that were treated. Although bone metastases are most common among breast and prostate carcinomas, in our study, the most common primary tumors were liver and lung, only 2 patients with breast cancer was included. This may be because that liver and lung cancer are common in China. By contrast, breast and prostate carcinomas are known to be radiosensitive, and most bone metastatic patients could achieve well pain control following EBRT, thus negating the need for further ^125^I implantation treatment. Secondly, this study is a single arm observational study, outcomes can be attributable to the intervention itself, a placebo effect, the natural course of the disease, or confounding by time-varying factors. In the future, a multicenter randomized controlled trial should be carried out to further test and to develop the curative effect of ^125^I seed brachytherapy for painful bone metastases after failure of EBRT.

In conclusion, we presented our experience of treating painful bone metastases after failure of EBRT by ^125^I seed implantation. It can improve both local control and quality of life, suggesting that ^125^I brachytherapy is a feasible and effective modality for the treatment of bone metastases in this population.
